# Nutritional Composition, Mineral Profiling, In Vitro Antioxidant, Antibacterial and Enzyme Inhibitory Properties of Selected Indian Guava Cultivars Leaf Extract

**DOI:** 10.3390/ph16121636

**Published:** 2023-11-21

**Authors:** Moses Sam Arul Raj, Singamoorthy Amalraj, Saud Alarifi, Mohan G. Kalaskar, Rupesh Chikhale, Veerasamy Pushparaj Santhi, Shailendra Gurav, Muniappan Ayyanar

**Affiliations:** 1 Department of Botany, A.V.V.M. Sri Pushpam College (Affiliated to Bharathidasan University), Poondi, Thanjavur 613 503, India; samarulraj98@gmail.com; 2 Division of Phytochemistry and Drug Design, Department of Biosciences, Rajagiri College of Social Sciences, Kalamaserry, Kochi 683 104, India; s.amalraj101@gmail.com; 3 Department of Zoology, College of Science, King Saud University, P.O. Box 2455, Riyadh 11451, Saudi Arabia; 4 Department of Pharmacognosy, R. C. Patel Institute of Pharmaceutical Education and Research, Shirpur 425 405, India; kalaskar.mohan@gmail.com; 5 UCL School of Pharmacy, 29-39 Brunswick Square, London WC1N 1AX, UK; r.chikhale@ucl.ac.uk; 6 Department of Horticulture, Anbil Dharmalingam Agricultural College and Research Institute, Tamil Nadu Agricultural University, Navalur Kuttappattu, Tiruchirappalli 620 027, India; 7 Department of Pharmacognosy, Goa College of Pharmacy, Goa University, Panaji 403 001, India; shailendra.gurav@nic.in

**Keywords:** antioxidants, enzyme inhibition, germplasms, pharmacology, proximate analysis, nutraceuticals

## Abstract

*Psidium guajava* L. is a small evergreen tree known for its magnificent medicinal and nutritional value. This study aimed to evaluate the nutritional profile and in vitro pharmacological potentialities of the different leaf extracts of four cultivars of *Psidium guajava* namely Surka chitti, Allahabad safeda, Karela, and Lucknow-49. The standard procedures of the Association of Official Analytical Chemists (AOAC) were followed to carry out the nutritional analysis and all of the cultivars recorded the presence of elements at a nominal range. The highest presence of phenols (125.77 mg GAE/g) and flavonoids (92.38 mg QE/g) in the methanolic leaf extract of the Karela cultivar was recorded. A wide range of minerals such as sodium, phosphorus, magnesium, zinc, and boron were recorded with a higher percentage in the Karela cultivar of *Psidium guajava*. In the enzyme inhibitory assays, Allahabad safeda showed potential inhibition with an IC_50_ of 113.31 ± 1.07, 98.2 ± 0.66 and 95.73 ± 0.39 μg/mL in α-amylase, α-glucosidase, and tyrosinase inhibition assays, respectively. The strong antioxidant effect was established by Lucknow-49 (IC_50_ of 74.43 ± 1.86 μg/mL) and Allahabad safeda (IC_50_ of 78.93 ± 0.46 μg/mL) for ABTS and DPPH assays, respectively. The ethyl acetate and methanolic leaf extracts of the Allahabad safeda cultivar showed better inhibition against *Pseudomonas aeruginosa* with an MIC of 14.84 and 28.69 µg/mL, respectively. A decent mean zone of inhibition was recorded in methanolic leaf extract that ranged from 21–25 mm in diameter against the tested bacterial strains (*Proteus vulgaris*, *Bacillus subtilis*, and *P. aeruginosa*). This is the first scientific report on the comparative and comprehensive analysis of indigenous guava cultivars to evidently shortlist the elite cultivars with enriched dietary nutrition and biological activities.

## 1. Introduction

Plants are considered to be vital natural resources of bioactive compounds that are capable of treating several ailments through different medicinal preparations. The scientific communities are engraving their knowledge over possible extractions of these bioactive phytocompounds. The phytochemicals from natural sources have been used as active components in drug formulations by the pharmaceutical and nutraceutical industries [1]. Deficiencies of essential nutrients, vitamins, and minerals cause severe impacts on the normal routine life of mankind.

An average of 53% of mortality is caused due to malnutrition and children below 5 years of age are mainly prone to it [2]. Hence, there is an emerging need to deliver essential nutrients through dietary supplements from economical sources. The demand for protein supplements is increasing exponentially day by day. Researchers and food industries are very keen in search of an alternative to animal protein to minimize the risk of health issues and to improve the nutritional quality of the food. Plant-based proteins are said to be feasible for all levels of income and without any negotiations in the dietary quality [3]. The validation of medicinally important phytocompounds solely relies on the process of extraction and the nature of the solvent system implemented. To optimize active extraction, solvents at different state of polarity is preferred by the researchers. It was revealed that the solvent penetrates the leaf material and solubilizes the phytocompounds which have the same polarity. There are also some other factors like temperature, the nature of solvent used, time, and composition of metabolites that influence the process of extraction [4].

Guava (*Psidium guajava* L.) is a small tree, evergreen in nature, belongs to the family Myrtaceae, and is regarded to be an important member of the clan. The plant has been reported to have originated in South American countries and then migrated to the rest of the world, especially to various tropical and subtropical countries [5]. It is widely grown and cultivated as a commercial fruit crop in tropical regions such as India, Bangladesh, Pakistan, Indonesia, and South America. All parts of the tree are considered to bear some medicinal properties and the fruits are edible and rich in nutrition. The cultivation of this commercial crop is highly feasible as it grows well in different edaphic climatic conditions [6]. It is not only harvested for its commercial value but also for its potential to cure an array of human illnesses.

The medicinal importance of leaves and fruits has been recorded since ancient times and is used in many folk medicinal practices. The various pharmacological activities endowed to this plant are antibacterial [7], antioxidant [8], anti-inflammatory and anti-allergic [9], antidiabetic [10], immunomodulatory [11], anti-melanogenesis [12], wound healing properties [13], anti-hypertension [14], and anti-cancer [15], etc.

Guava leaves are considered to be very important as most of the secondary metabolites accumulate in this part. The leaves of the guava are rich in aroma when crushed and with enriched phenolic compounds that help to modulate blood sugar levels and various other health issues [16]. The reason behind this is because of the secretory structures present over the surface and internal parts of the leaf. This structure promotes the synthesis of various bioactive compounds like polyphenols, naringenin, rutin, gallic acid, catechin, kaempferol, epicatechin, isoflavonoids, and flavonoids such as quercetin and guaijaverin, and all of these bioactive compounds are highly responsible for antioxidant, antimicrobial, and anti-inflammatory activities [8,17,18,19]. Several chronic diseases like gastroenteritis, diarrhea and dysentery, wounds, ulcers, arthritis, toothache, anorexia, cholera, pulmonary disorders, laryngitis, and skin disorders can be treated through the bioactive phytocompounds that are present in the different parts of the guava [20].

Recently, benzene-1,2-diol,2′-O-methyl guanosine, 5-bromo-8-(5-nitrosalicylidene amino) quinoline hydrochloride, guavanoic acid, protocatechuic acid, oleanolic acid, and 2α-hydroxyursolic acid, which favored the antioxidant and antidiabetic activity in the aqueous extract of *P. guajava* was reported [10]. Also, a few studies have reported that the superior antidiabetic activity in the *P. guajava* leaf was due to the presence of phenolics, flavonoids, and pentacyclic triterpenoids [1,21,22]. The ethanolic leaf extract of *P. guajava* was reported to be rich in ellagic acid, hyperin, guajaverin, reynoutrin, genistin, rutin, avicularin, quercitrin, kaempferol, quercetin, maslinic acid, corosolic acid, ursolic acid, and oleanolic acid [23] along with three novel compounds, namely, psidials L, psidials M, and psipinene [24]. Also, the methanolic leaf extract of *P. guajava* exhibited the presence of 51 phytocompounds with major compounds like quinic acid, catechin, casuarinin, phlorizin, quercetin, tricin, and guavinoside A [25].

The existing literature supports the pharmacological uses of *Psidium guajava*, but only a few reports have been made on comparative studies on the biological potentialities of different Indian cultivars. Therefore, this current study is focused on the investigation of biological efficiencies of different Indian cultivars of *Psidium guajava* leaf extracts.

## 2. Results and Discussion

Several nutraceutical industries are working furiously to meet the demands of mankind for balanced nutrition from natural sources. Guava is considered the fifth most widely cultivated commercial crop which is said to possess vital minerals and vitamins. About 30 different varieties/cultivars of guava (*Psidium guajava* L.) are available and cultivated in different states of India for their medicinally important and nutritious fruits [8]. In this present study, the guava cultivars viz., Surka chitti, Allahabad safeda, Karela, and Lucknow-49 were chosen for elemental analysis and exploration of their nutritional composition, followed by a study of their antidiabetic activity, free radical scavenging potential, anti-tyrosinase effect, and antibacterial activities.

The yield of the extracts is greatly influenced by the polarities of solvents used for the extraction process. The yield percentage of the leaf extracts of different guava cultivars ranged from 0.64 to 2.32%. The highest percentage (2.32%) was recorded with the aqueous extract of the Surka chitti cultivar. The results of the present study were in accordance with Rehan et al. [26] who reported a 2.5% yield in aqueous extract of guava leaf. In contrast, Jani et al. [27] recorded the highest yield of 9.65% in methanolic extract of guava leaf. It was revealed that polarity and total extract yield are directly proportional.

### 2.1. Mineral Profiling and Nutritional Value of the Leaf Extracts of Guava Cultivars

The selected cultivars are regarded to be very popular varieties in India as well as in other tropical regions of the world with enriched total soluble solids, soluble proteins, ascorbic acid, and various other essential elements [28]. The mineral contents viz., N, P, K, Ca, Na, Mg, B, Zn, Cu, and Fe of different guava cultivars were estimated and are presented in Table 1.

The total nitrogen content ranged from 1450 to 2800 mg/100 g with the Surka chitti cultivar showing the highest nitrogen content while Karela expressed the lowest. The highest total iron and boron content was observed in the Allahabad safeda cultivar with the values 51.6 and 40 mg/100 g, respectively. Elements such as Ca, Mg, Na, and Zn were found to be in higher ratios in the Karela cultivar of *P. guajava* with 2720, 1440, 24, and 0.48 mg/100 g, respectively. The Lucknow-49 cultivar is said to possess a wide range of elements such as P, Cu, and K with values of 320, 0.663, and 1070 mg/100 g, respectively, with greater composition. The highest total potassium content was observed with the Surka chitti cultivar, while the lowest was observed in the Allahabad safeda cultivar with values ranging from 480 to 1140 mg/100 g. Previously, Thomas et al. [29] reported the presence of elements such as calcium (1660 mg/100 g), iron (13.50 mg/100 g), magnesium (440 mg/100 g), phosphorus (360 mg/100 g), and potassium (1602.0 mg/100 g) in guava leaves which was moderately lower than our results.

**Figure 1:**
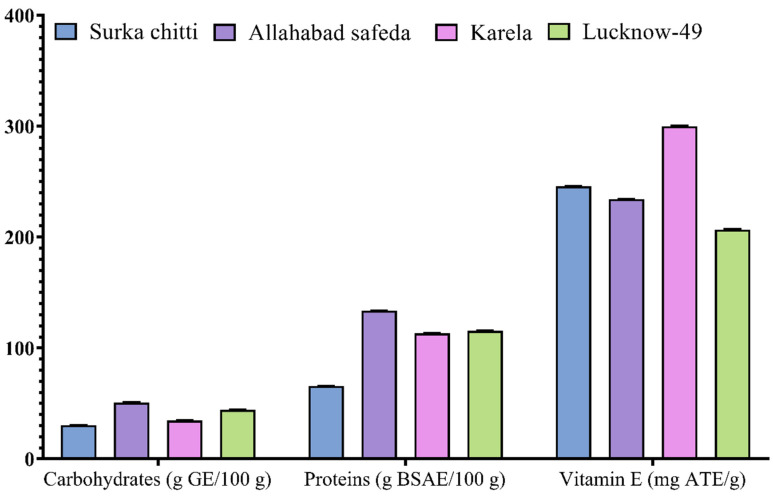
Proximate analysis of leaf extracts obtained from different guava cultivars. Values are specified as mean (n = 3) ± standard deviation.

The proximate analysis was estimated for the selected four guava cultivars and the results are shown in Figure 1. Total carbohydrate content ranged from 30.56 to 50.89 g GE/100 g. The highest carbohydrate content was recorded in the Allahabad cultivar (50.89 g GE/100 g) and the Lucknow-49 cultivar also showed moderate results with 44.33 g GE/100 g. The total protein content of guava cultivars was greater than carbohydrates and it ranged from 65.87 to 133.51 g BSAE/100 g. Like carbohydrates, the Allahabad safeda cultivar (133.51 g BSAE/100 g) also expressed higher protein content followed by Lucknow-49 (115.49 g BSAE/100 g), Karela (113.3 g BSAE/100 g), and Surka chitti (65.87 g BSAE/100 g).

Total vitamin E content was estimated for all the guava cultivars and the Karela cultivar established a higher total vitamin E content with 299.96 mg ATE/g followed by Surka chitti (245.84 mg ATE/g), Allahabad safeda (234.27 mg ATE/g), and Lucknow-49 (206.82 mg ATE/g). To support our study, Rehman et al. [30] reported a protein composition of 0.1 to 0.6 mg /100 g in guava leaf extracts. Likewise, Shabbir et al. [31], recorded 12.74% of carbohydrates in guava leaf extracts which was relatively lower than our results. Bhadkaria et al. [32] studied the nutritional composition of *Vigna aconitifolia* (moth bean) and reported that the total protein content ranged from 11.30 ± 1.02 to 30.74 ± 1.95 mg/g.

**Figure 2:**
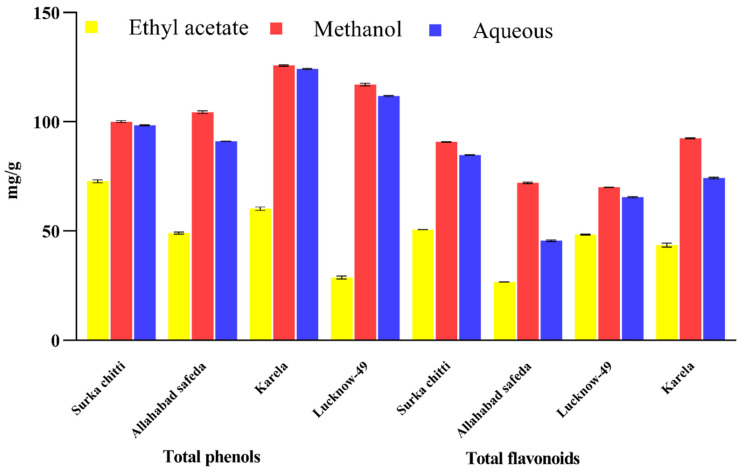
Quantitative estimation of total phenols and flavonoids in the leaf extracts of different *P. guajava* cultivars. Values are specified as mean (n = 3) ± standard deviation.

The total phenolics (TPC) and total flavonoids (TFC) of the leaf extracts of different guava cultivars are represented in a graphical illustration (Figure 2). The TPC of the extract was measured in gallic acid equivalents. The TPC of different guava cultivars ranged from 28.72 to 125.72 mg GAE/g with the highest quantity in methanolic leaf extract of Karela cultivar with 125.77 mg GAE/g, followed by Lucknow-49 (117.05 mg GAE/g), Allahabad safeda (104.41 mg GAE/g) and Surka chitti (100.11 mg GAE/g). All of the leaf extracts in the studied guava cultivars were also subjected to the estimation of total flavonoids and were measured in quercetin equivalents.

As with TPC, the methanolic extract dominated over the other extracts for flavonoid content. The methanolic leaf extract of the Karela cultivar showed a greater amount of flavonoids with 92.38 mg QE/g and the lowest quantity was observed in the ethyl acetate leaf extract of the Allahabad safeda cultivar with 29.66 mg QE/g. Kumar et al. [1] stated that phenolic compounds present in the guava leaves are the vital bioactive compounds responsible for the distinguished antioxidant and hypoglycemic activities. The total phenols and flavonoid content of the methanolic leaf extract of guava were 85.36 ± 1.89 mg GAE/g and 76.5 ± 10.41 mg QE/g, respectively, as reported by Bakshi et al. [33] whereas our results with methanol extract had topped high. Likewise, Amalraj et al. [34] estimated the total phenolics and flavonoid content of different extracts of *Canthium coromandalicum* leaf and recorded that hydroalcoholic extract exhibited the highest phenolic content with 58.03 mg GAE/g extract, which was much lower compared to our results. Krupa et al. [35] also reported that the methanolic extract of *Moringa concanensis* seeds showed the highest TPC with 76.41 mg GAE/g, which was also lower than our results. Similar to this, Mazumder et al. [36] studied the TPC of guava leaf and reported 66.00 ± 1.21 mg GAE/g which was relatively lower than the results of the present study.

### 2.2. In Vitro Biological Studies of Guava Leaf Extracts

#### 2.2.1. Enzyme Inhibitory Effects of the Guava Cultivars

**Figure 3:**
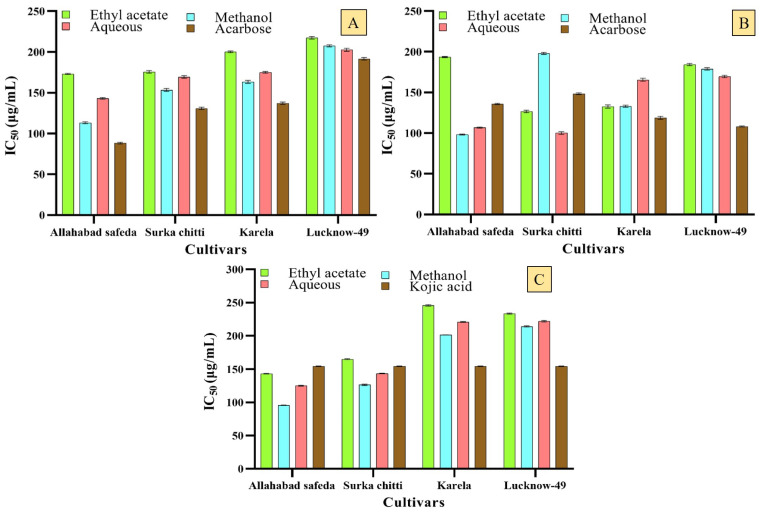
In vitro enzyme inhibitory ((**A**)—α-amylase; (**B**)—α-glucosidase; (**C**)—tyrosinase enzyme inhibitory assays) activities of different guava cultivar leaf extracts. Values are represented as IC_50_ and mean (n = 3) ± standard deviation.

The guava is predominantly known for its antihyperglycemic activity and the leaf and bark extracts of this plant are used by ethnic people traditionally from the regions of South Africa, Indonesia, Madagascar, etc. to treat diabetes in different medicinal formulations such as raw juice, decoctions, and infusions [37]. It was reported that the flavonoid compounds and polysaccharides existing in the guava leaves are regarded as the responsible constituents for their promising hypoglycemic activity by inhibiting the activity of α-amylase and α-glucosidase enzymes [1]. The results of the in vitro antidiabetic activity were depicted as graphical charts as revealed by α-amylase and α-glucosidase enzyme inhibition assays (Figure 3A,B).

The methanolic leaf extracts of Allahabad safeda, Surka chitti, and the Karela cultivars expressed moderate α-amylase enzyme inhibitory effect with an IC_50_ of 153.48 ± 1.75, 113.31 ± 1.07, and 163.50 ± 1.90 μg/mL, respectively. The tremendous inhibition of the α-glucosidase enzyme was observed with methanolic leaf extracts of Surka chitti and Allahabad safeda cultivars with an IC_50_ of 100.09 ± 1.58 and 98.2 ± 0.66 μg/mL, respectively, and they were comparatively better than the standard Acarbose (148.43 ± 0.97 and 106.85 ± 0.68 μg/mL). Previously, the essential oils obtained from the guava cultivars, Lucknow-49 (220.93 ± 0.81 µg/mL), Allahabad safeda (237.8 ± 0.71 µg/mL), and Surka chitti (239.50 ± 0.33 µg/mL) exhibited notable enzyme inhibitory effects against the α-amylase and α-glucosidase enzymes [8]. According to a recent study, the alcoholic extract of guava leaf showed better results with an IC_50_ of 0.5 ± 0.01 μg/mL in α-glucosidase assay and 10.6 ± 0.4 μg/mL with the ethanolic bark extracts over α-amylase enzyme inhibitory activity [38].

It was revealed that the efficient antidiabetic effects of guava leaves are due to the phenolic compounds present in the extract [10]. The present study also reveals the same with higher phenolic content in methanolic extract which is attributed to the better enzyme inhibitory potential. The prevalence of polyphenols (1717 ± 6.43 mg GAE/g) aids in the reduction of blood glucose levels by promoting the secretion of insulin and regulating the level of glucose from the liver [31]. The flavonoids extracted from the guava also directly influenced hyperglycemia by lowering fasting plasma glucose and insulin resistance in diabetes-induced mice [39]. Polyphenolic compounds like catechin, naringenin, and quercetin and their derivatives, isoquercetin and guaijaverin are confined to guava leaves with greater viability in lowering blood sugar levels [40].

The anti-hyperpigmentation analysis was performed for the leaf extracts of different guava cultivars (Figure 3C). The results revealed that the extract has expressed a nominal inhibitory effect for the anti-hyperpigmentation activity. The methanolic leaf extracts of Allahabad safeda and Surka chitti cultivars revealed higher tyrosinase inhibition with an IC_50_ of 95.73 ± 0.39 and 126.52 ± 1.15 µg/mL, respectively. Likewise, Raj et al. [8] described the tyrosinase enzyme inhibitory activity of essential oil of different guava cultivars, and the highest inhibition was recorded with an IC_50_ of 133.45 µg/mL for the Karela cultivar and it was relatively lesser than the results observed in the present report. Suroowan et al. [41] analyzed the tyrosinase enzyme inhibitory effect of the *Tambourissa peltata* leaf and revealed that the methanolic extract (155.30 ± 0.55 mg KAE/g) had a better inhibition effect among ethyl acetate and distilled water extracts and this also supports the results of the present study that recorded the highest percentage of inhibitions in methanolic extracts.

#### 2.2.2. Free Radical Scavenging Potential of Guava Cultivars

**Figure 4:**
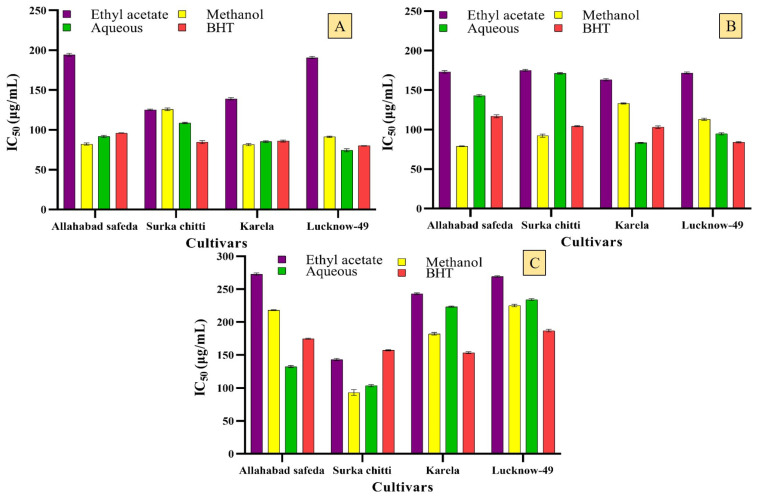
In vitro antioxidant ((**A**)—ABTS; (**B**)—DPPH; (**C**)—Superoxide free radical scavenging assays) activities of the leaf extracts of different guava cultivars. Values are represented as IC_50_ and mean (n = 3) ± standard deviation.

There is a demand for natural sources of antioxidants without any side effects and these natural sources from plants must be a potential. With this in mind, the different cultivars of guava leaf extracts were analyzed for their antioxidant potential. In ABTS radical cation decolorization assay, the methanolic extracts of the cultivars, Allahabad safeda (IC_50_ of 82.23 ± 1.35 µg/mL) and Karela (81.50 ± 1.38 µg/mL) expressed notable results when compared with the standard BHT which showed an IC_50_ of 95.99 ± 0.34 and 85.93 ± 1.20 µg/mL, respectively) (Figure 4A). Aqueous extracts of Lucknow-49 and Surka chitti showed better values with an IC_50_ of 74.43 ± 1.86 and 108.76 ± 0.91 µg/mL, respectively.

The DPPH radical scavenging potential of the tested extracts showed greater results when compared to the standard (Figure 4B). The Surka chitti and Allahabad safeda cultivars showed higher scavenging potential in methanolic extract with an IC_50_ of 92.30 ± 1.94 and 78.93 ± 0.46 µg/mL, respectively. The aqueous extract of Karela and Lucknow-49 cultivars also revealed good scavenging activity with an IC_50_ of 83.22 ± 0.66 and 94.60 ± 1.29 µg/mL, respectively. The results of superoxide radical scavenging activity are represented in Figure 4C wherein the methanolic leaf extract of the guava cultivars showed viable scavenging potential against the superoxide radical with Surka chitti, Karela, and Lucknow-49 as the most promising examples.

A strong scavenging potential was recorded in the aqueous extract of the Allahabad safeda cultivar with an IC_50_ of 132.68 ± 1.53 µg/mL which was better than the standard BHT (IC_50_ of 174.94 ± 0.70 µg/mL). Jassal et al. [42] reported that the IC_50_ of DPPH radical scavenging of guava leaf essential oil was 445 ± 0.001 µg/mL which was lower than our results. Dewage et al. [43] studied the antioxidant activity of the guava leaf through DPPH assay and the resultant IC_50_ was 90.07 ± 0.73 µg/mL and when compared to our results with methanolic extract (78.93 ± 0.46 µg/mL) it was very low. Similarly, Jani et al. [27] recorded the DPPH radical scavenging capacity of *Psidium guajava* with an IC_50_ of 45.52 µg/mL. The increase in concentration of phenolic compounds empowers greater radical scavenging of the tested extracts by enhancing the donation of hydrogen atoms to the unpaired electrons of the free radicals [44]. Ruksiriwanich et al. [45] also studied the antioxidant activity of the ethanolic leaf extract of *P. guajava* through DPPH and ABTS radical scavenging activities and recorded the results with 444.05 ± 1.01 mg TE/g extract for DPPH and 424.80 ± 31.05 mg TE/g extract for ABTS. Purba et al. [46] confirmed that the leaves of *P. guajava* are enriched with several health-promoting efficiencies and also render the reactive oxygen species by formulating several antioxidant phytochemicals like p-coumaric acid, sinapic acid, ferulic acid, myricetin, eugenol, kaempferol, etc.

#### 2.2.3. Antibacterial Activity of Guava Leaf Cultivars

**Figure 5:**
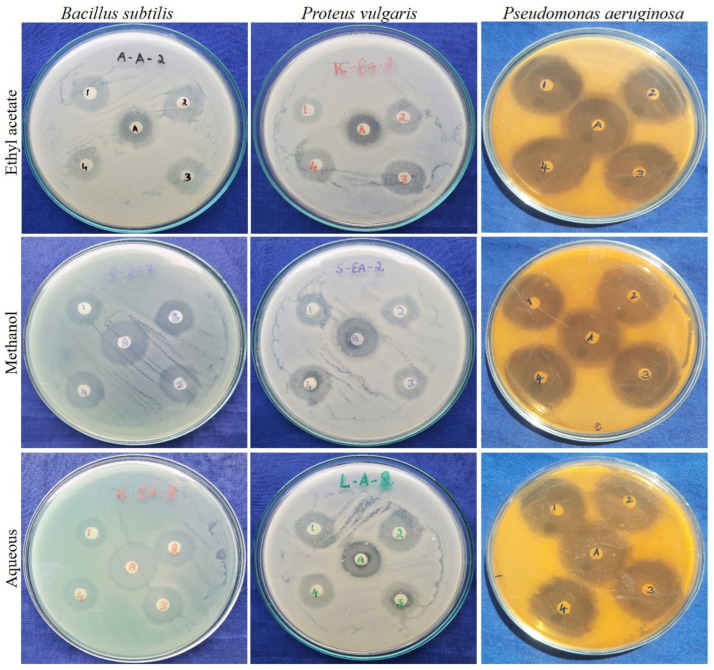
Zone of inhibition expressed by the leaf extracts of different *P. guajava* cultivars against the growth of selected bacterial strains. The numbers 1, 2, 3, and 4 in the plates represent the cultivar names, Surka chitti, Allahabad safeda, Karela, and Lucknow-49, respectively. A—Antibiotic. Values are specified as mean (n = 3) ± standard deviation.

Antibacterial agents from natural sources have been prevalent for decades, but still, some microbial strains are resistant to certain antibiotics and cause a major threat to mankind. To resolve this, the present study was conducted in order to scrutinize the leaf extracts of different guava cultivars for their antibacterial efficacy. The tested bacterial strains, *P. vulgaris*, *B. subtilis*, and *P. aeruginosa* were susceptible to the tested leaf extracts of guava cultivars, Surka chitti, Allahabad safeda, Karela, and Lucknow-49 and all of these cultivars expressed better activity and were comparable with the standard antibiotic used in this study (Figure 5). Among the solvents used, methanolic and aqueous leaf extracts of the guava cultivars showed better results than the ethyl acetate extract. The highest mean zone of inhibition was recorded with the methanolic leaf extract of all of the cultivars against the bacterial strains *B. subtilis*, *P. vulgaris*, and *P. aeruginosa,* and the mean zone of inhibition ranged from 21.66 ± 1.15 to 31.66 ± 0.57 mm.

The Lucknow-49 cultivar established potential antibacterial activity in a methanolic extract with the mean zone of inhibition of 24.66 ± 1.52, 25.66 ± 0.57, and 31.66 ± 0.57 mm against the growth of *P. vulgaris*, *B. subtilis*, and *P. aeruginosa*, respectively. Likewise, Naili et al. [47] reported that the bacterial strain, *B. subtilis* was susceptible to methanolic extracts of guava leaf with the mean zone of inhibition ranging from 13 to 17 mm, and their reported activity is regarded to be much lower than the present study. Almulaiky et al. [48] reported that the maximum zone of inhibition of the Aldabab and Anaa cultivars of guava against the bacterial strain, *P. aeruginosa* was 15 ± 1.6 and 18 ± 1.3 mm, respectively, and the results of the present study showed higher zone of inhibition with 25.66 ± 0.57 mm with the same bacterial strain.

Previously, the antibacterial activity of the methanolic leaf extract of guava was reported with the mean zone of inhibition ranging from 8.27 ± 0.44 to 12.3 ± 0.78 mm [4] whereas in the present study, the mean zone of inhibition ranged from 17.67 ± 0.57 to 25.66 ± 0.57 mm which is comparatively higher than the previous reports. To support our study, Pereira et al. [49] reported that the methanolic leaf extract of *P*. *guajava* had greater antibacterial activity against Gram-positive and Gram-negative bacterial strains and recorded the highest zone of inhibition with 21.0 ± 2.0 mg/mL against the bacterial strain *Streptococcus* beta-hemolytic.

The minimum inhibitory concentration (MIC) values of the leaf extracts of chosen guava cultivars showed decent efficacy, albeit sparsely. The MIC of the leaf extracts of different guava cultivars ranged from 3.71 to 950 µg/mL against the studied bacterial strains, *P. vulgaris, B. subtilis*, and *P. aeruginosa* (Table 2). Among the tested bacterial strains, *B. subtilis* and *P. aeruginosa* were susceptible to the studied extracts. The ethyl acetate and methanolic leaf extracts of the Allahabad safeda cultivar showed better inhibition against *P. aeruginosa* with MIC values of 14.84 and 28.69 µg/mL, respectively. The bacterial strain *B. subtilis* was highly susceptible to the methanolic leaf extract of the Lucknow-49 cultivar (3.71 µg/mL). The bacterial strain *P. vulgaris* showed partial susceptibility to all the tested leaf extracts of the guava cultivars. In contrast, Almulaiky et al. [48] studied the antibacterial activity of two cultivars of *P. guajava* and reported that the minimum inhibitory concentration of the Aldabab and Anaa cultivars against the bacterial strain *P. aeruginosa* were 1670 and 987 µg/mL, respectively, whereas the present study revealed better efficiency than this result over the same bacterial strain.

### 2.3. Correlation between the Quantitatively Analyzed Phytochemicals and Biological Assays

**Figure 6:**
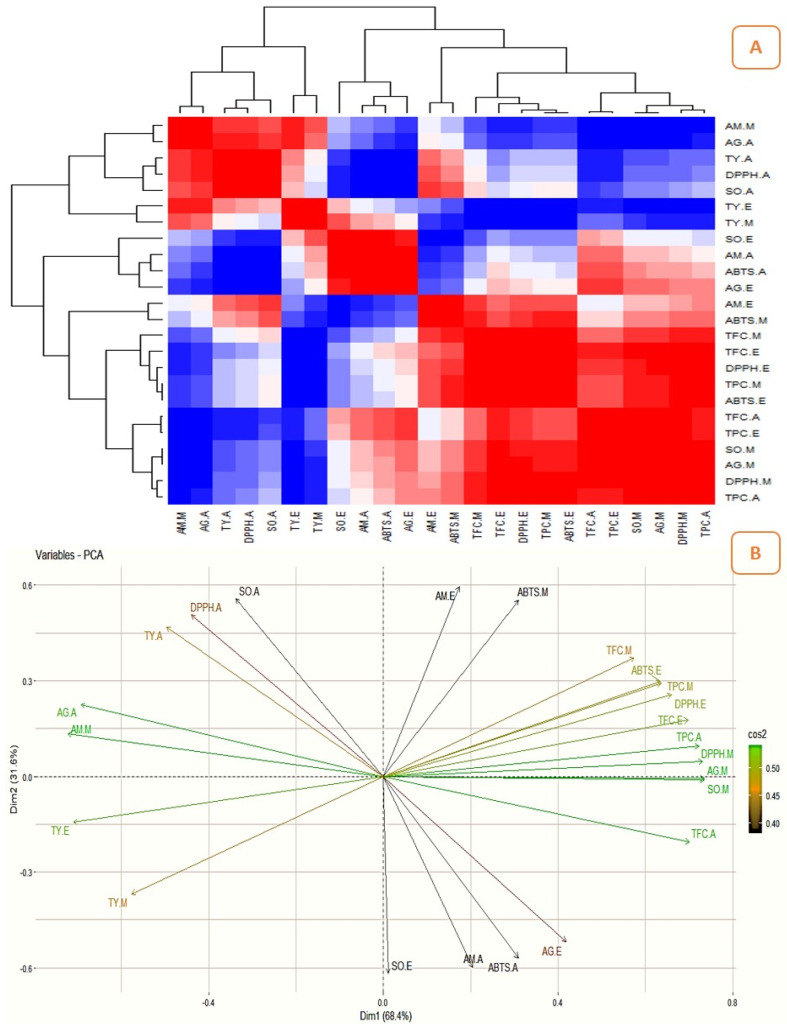
Correlation analysis and PCA for guava cultivar, Surka chitti. (**A**)—Heatmap illustrating the relationship between the phytochemicals and their biological activities (the dark red and dark blue indicates enhanced correlation); (**B**)—Biplot principal component analysis (PCA) of phytocompounds and their biological activities.

**Figure 7:**
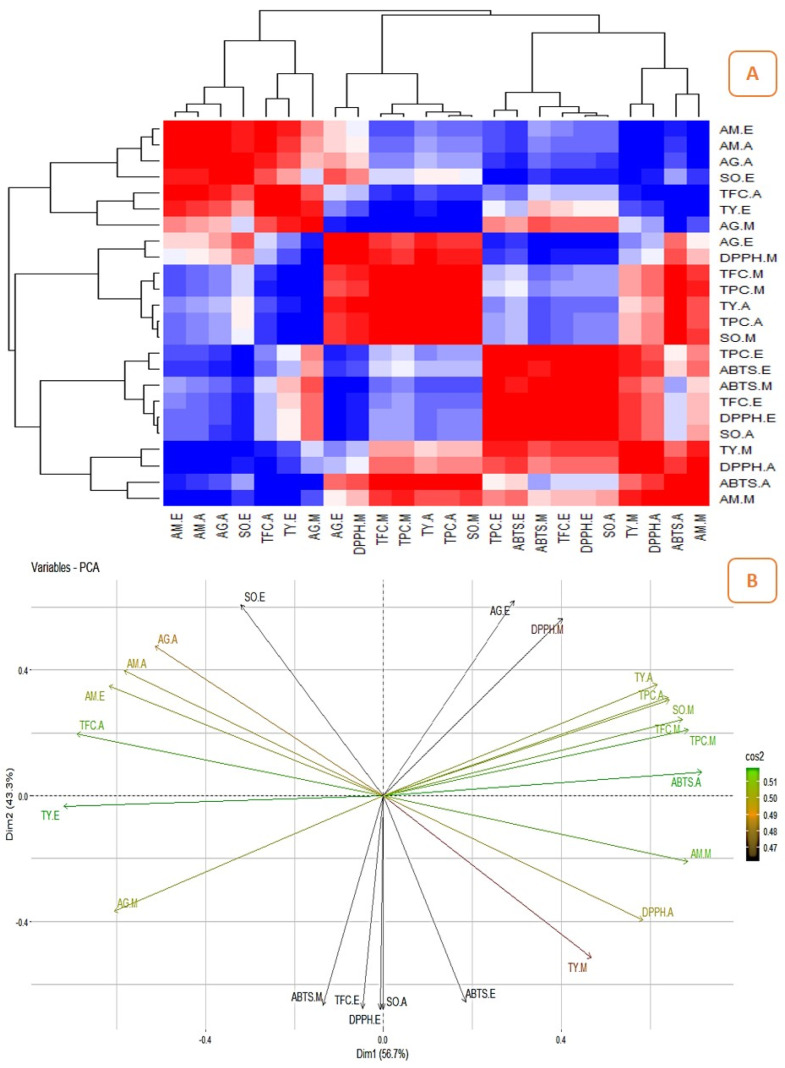
Correlation analysis and PCA for guava cultivar, Allahabad safeda. (**A**)—Heatmap illustrating the relationship between the phytochemicals and their biological activities (the dark red and dark blue indicates enhanced correlation); (**B**)—Biplot principal component analysis (PCA) of phytocompounds and their biological activities.

**Figure 8:**
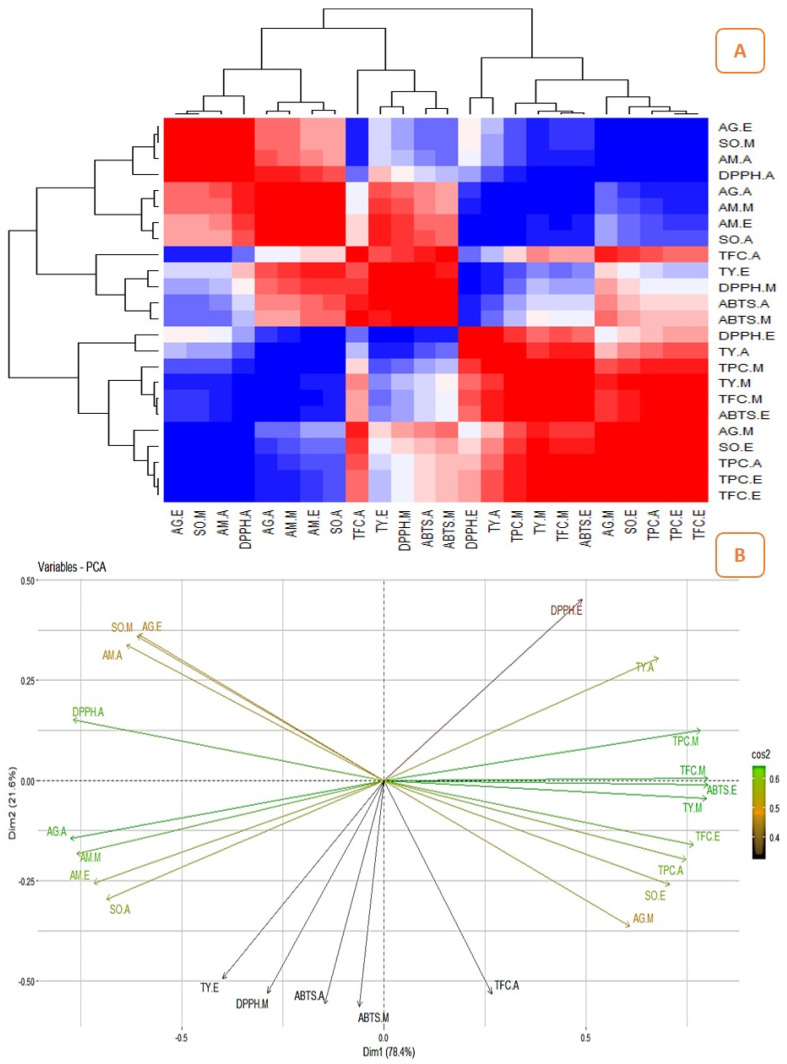
Correlation analysis and PCA for guava cultivar, Karela. (**A**)—Heatmap illustrating the relationship between the phytochemicals and their biological activities (the dark red and dark blue indicates enhanced correlation); (**B**)—Biplot principal component analysis (PCA) of phytocompounds and their biological activities.

**Figure 9:**
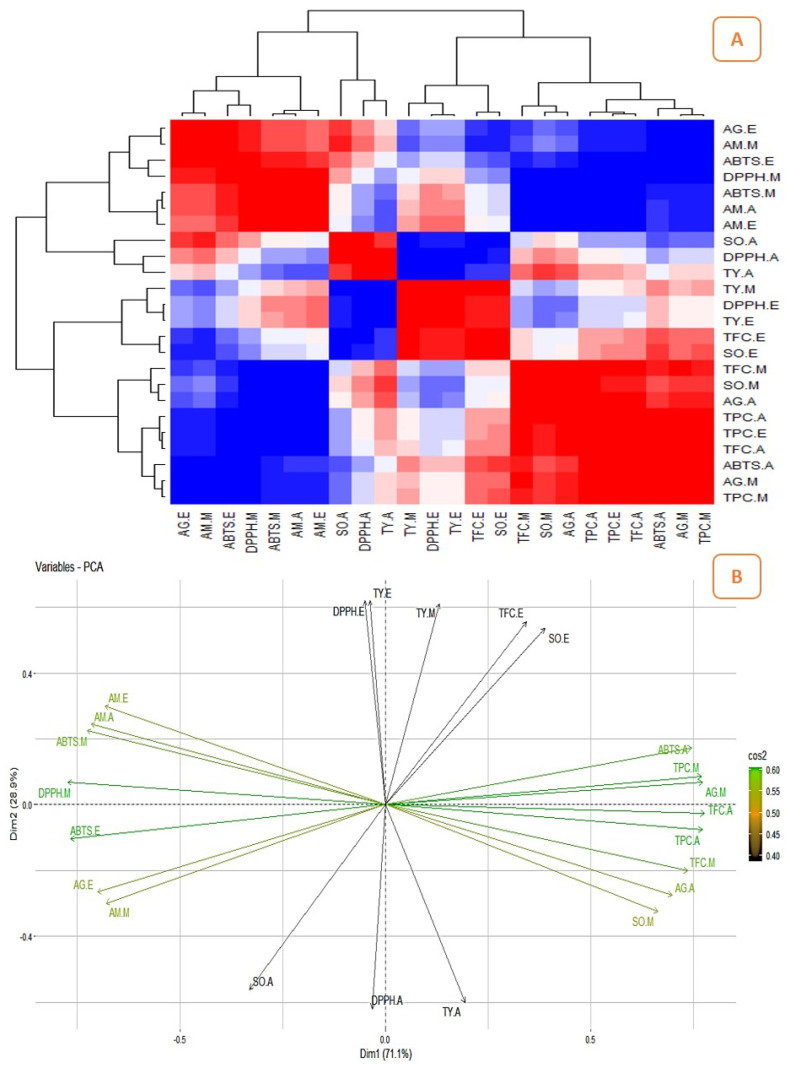
Correlation analysis and PCA for guava cultivar, Lucknow-49. (**A**)—Heatmap illustrating the relationship between the phytochemicals and their biological activities (the dark red and dark blue indicates enhanced correlation); (**B**)—Biplot principal component analysis (PCA) of phytocompounds and their biological activities.

The correlation studies revealed the relationship between the pharmacological activities and phytochemicals and their influence on biological activities (Figure 6A, Figure 7A, Figure 8A and Figure 9A). The different colours in the image were associated with the amount of phytochemicals which correlated with the antioxidant and enzyme inhibitory properties. The dark red and dark blue indicates higher correlation, mild red and mild blue represent moderate correlation and light blue and light red shows lower correlation. The results showed that the phenolic content of the Surka cultivar strongly correlates with the ABTS (r^2^ = 0.82), DPPH (r^2^ = 0.93), SO (r^2^ = 0.96), and α-glucosidase (r^2^ = 0.95) assays. The free radical scavenging activity of ABTS (r^2^ = 0. 95; r^2^ = 0. 99), DPPH (r^2^ = 0.79; r^2^ = 0.82), and SO (r^2^ = 0.99; r^2^ = 0.86) strongly correlates with TPC and TFC in the Allahabad cultivar. The aqueous extract of TPC and TFS show a greater and significant (*p* < 0.05) positive correlation with the tyrosinase and α-amylase enzyme assays, respectively. The TFC of the ethyl acetate extract showed a strong and significant positive correlation with the antioxidant effects of ABTS (r^2^ = 0.99), DPPH (r^2^ = 0.35), and SO (r^2^ = 0.98) in the leaf extracts of the Karela cultivar, and a notable correlation of TPC with SO (r^2^ = 0.98) and tyrosinase (r^2^ = 0.22) was also observed. In the Lucknow-49 cultivar, the TPC was positively correlated with ABTS (r^2^ = 0.99) and α-glucosidase (r^2^ = 0.93) enzymes. The TFC shows a strong and significant positive correlation with antioxidant assays like ABTS (r^2^ = 0.95) and SO (r^2^ = 0.98), and tyrosinase enzyme inhibition assay (r^2^ = 0.96). The present findings show that the polyphenolic and flavonoid compounds found in leaf extracts have a significant impact on pharmacological activity.

### 2.4. Principal Component Analysis (PCA)

The graphical representation of PCA demonstrated the relationship between the phytocompounds of guava cultivars and pharmacological assays. Figure 6, Figure 7, Figure 8 and Figure 9 show the relationships and correlation between the variables like phytocompounds and their biological activities, as well as the principal components of the data. In the Surka chitti cultivar, the data variance was distinguished by two factors, dimension 1 with 68.4% and dimension 2 with 31.6% (Figure 6B). The obtained illustration revealed that ABTS, DPPH, α-amylase, and α-glucosidase assays were positively correlated with TPC and TFC. The data variance in the Allahabad safeda was distinguished by dimension 1 at 56.7% and dimension 2 at 43.3% (Figure 7B).

The PCA also reveals that ABTS, DPPH, SO, α-glucosidase, and tyrosinase assays were positively correlated with TPC and TFC. In the Karela cultivar, the data variance was separated by two factors such as dimension 1 with 78.4% and dimension 2 with 21.6% (Figure 8B). The obtained data revealed that DPPH, SO, α-amylase, α-glucosidase, and tyrosinase enzyme assays were positively correlated with TPC and TFC. Lucknow-49 showed data variance with two different factors that were dimension 1 with 71.1% and dimension 2 with 28.9% (Figure 9B). The graphical representation also illustrates that assays like ABTS, DPPH, α-glucosidase, and α-amylase recorded a positive correlation with the TPC. The yield pattern was studied by correlating with climatic conditions and reported higher yield during the rainy season in some species of *Psidium* [50]. Yoon and Kim [51] correlated the metabolites and antidiabetic, antioxidant, and antidementia activities of fermented extracts of *Artemisia capillaris* and reported that the variations in the antioxidant activity were altered by the polyphenolic compounds. The correlation between the influence of plant parts and origin on the chemical composition of *Rosa arvensis* revealed that the plant part is more important for its composition than the locality of the plant’s origin [52]. This is the first report on the correlation studies between the phytoconstituents and biological efficiencies of different guava cultivars.

## 3. Materials and Methods

### 3.1. Collection and Preparation of Guava Leaf Extracts

Fresh leaves of *P. guajava* cultivars such as Allahabad safeda, Karela, Lucknow-49, and Surka chitti were collected from the experimental plots of the Horticultural College and Research Institute for Women (Tamil Nadu Agricultural University, Tiruchirappalli, India), Tiruchirappalli, India. The leaves were washed under running tap water to remove the external debris and dust deposits on the surface of the leaf. The washed leaves were then shade-dried in a clean environment and ground to powder. The extract was prepared from the grounded powder using three solvents with different polarities namely, ethyl acetate, methanol, and aqueous. The extracts were then filtered through Whatman No.1 filter paper and concentrated under reduced pressure (at 40 °C) until the formation of solid residues. The obtained extracts were then dried to remove excess solvents, if any, and kept at 4 °C for further studies. The yield percentage of extracts was calculated through the formula:

Yield (%) = mass of the extract obtained/initial mass of the leaf sample × 100

### 3.2. Nutritional Profiling

#### 3.2.1. Elemental Analysis

The nutritional factors of different guava cultivars’ leaf extracts were examined by the previously described standard procedures [53]. The composition of minerals and micronutrients (Cu, Fe, Mn, and Zn) in the extracts was solved using atomic absorption spectroscopy (AAS). The estimation of phosphorus content in the extract was obtained through the vanado-molybdate method, while potassium and sodium were evaluated using a flame photometer as described in AOAC [53].

#### 3.2.2. Proximate Analysis

The total carbohydrate content was determined through the Anthrone method [54]. The resultant values are expressed as g glucose equivalent/100 g. The total protein content was estimated using Lowry’s method [54] and the results were expressed using g bovine serum albumin equivalent/100 g. The total vitamin E content was determined using the previously described methods of Parimelazhagan [54]. The results are expressed as mg α-tocopherol equivalent/g. The total phenolic content (TPC) was measured using the Folin–Ciocalteu spectrophotometric method by following the previously described procedure of Amalraj et al. [55]. For the determination of TPC, gallic acid was used as the standard to prepare the reference curve, and the results were expressed in gallic acid equivalents (mg GAE/g). The total flavonoid content (TFC) was measured by the aluminium chloride (AlCl_3_) colorimetric method by following the protocols of Amalraj et al. [55] using quercetin to establish a standard curve and the resultant values were expressed as quercetin equivalents (mg QE/g).

### 3.3. Free Radical Scavenging Activity of Guava Leaf Extracts

#### 3.3.1. DPPH Radical Scavenging Assay

The DPPH radical scavenging ability of the leaf extracts was determined by the previously described procedures of Gangapriya et al. [56]. Butylated hydroxytoluene (BHT) was used as a positive control to determine the scavenging capacity of different cultivars of *P. guajava* leaf extracts. The calculated values of the percentage of inhibition of free radical scavenging ability were also expressed as IC_50_ values and the scavenging activity was calculated using the following equation:

% Inhibition = [(Acontrol − Asample)/Acontrol] × 100

#### 3.3.2. ABTS Radical Cation Decolorization Assay

The free radical scavenging capacity of guava leaf extracts was scrutinized by (2,2’-azino-bis (3-ethylbenzothiazoline-6-sulfonic acid)) (ABTS) radical cation decolorization assay [49] with BHT as a reference drug. The calculated values were denoted as IC_50_ values and the scavenging capacity of the samples was calculated using the following equation:

% Inhibition = [(Acontrol − Asample)/Acontrol] × 100

#### 3.3.3. Superoxide Radical Scavenging Activity

The efficacy of guava leaf extracts of four cultivars was screened for superoxide anion scavenging activity through the nitroblue tetrazolium (NBT) reduction method [56]. BHT was used as standard and the superoxide radical scavenging effect of leaf extracts was calculated with the following equation:

% Inhibition = [(Acontrol − Asample)/Acontrol] × 100

### 3.4. Enzyme Inhibitory Activity of Guava Leaf Extracts

#### 3.4.1. α-Amylase Enzyme Inhibitory Assay

The α-amylase enzyme inhibitory (porcine pancreatic α-amylase enzyme) assay was determined by following the protocol of Amalraj et al. [34] with acarbose as a reference indicator. The percentage of inhibition was calculated using the following equation:

% Inhibition = [(Acontrol − Asample)/Acontrol] × 100

#### 3.4.2. α-Glucosidase Enzyme Inhibitory Assay

The α-glucosidase enzyme inhibitory (p-nitro phenyl-α-d-glucopyranoside) assay was carried out by following the previously described method of Amalraj et al. [34] with acarbose as a standard drug. The percentage of inhibition was calculated using the following equation:

% Inhibition = [(Acontrol − Asample)/Acontrol] × 100

#### 3.4.3. Tyrosinase Enzyme Inhibitory Assay

The inhibitory effects of different cultivars of guava leaf extract over tyrosinase enzyme were tested by following the previously described procedures of Angelis et al. [57] with kojic acid as a reference indicator. The percentage inhibition was calculated using the equation:

% Inhibition = [(Acontrol − Asample)/Acontrol] × 100

### 3.5. Antibacterial Activity Leaf Extracts

#### 3.5.1. Zone of Inhibition (ZOI) by Disc Diffusion Method

The antibacterial activity of guava leaf extracts was determined through the disc diffusion method by following the previously described procedures of Borah et al. [58]. *Proteus vulgaris* (MTCC 7299), *Bacillus subtilis* (MTCC 3055), and *Pseudomonas aeruginosa* (MTCC 7093) are the bacterial strains used to analyze the antibacterial activity and the bacterial strains were purchased from the Microbial Type Culture Collection (MTCC), CSIR institute, Chandigarh, India. Amoxicillin and ciprofloxacin were used as standard antibiotic drugs as the Gram-negative bacterial strain *P. aeruginosa* is intrinsically resistant to the antibiotic, amoxicillin. The concentration of test samples and standards were standardized and fixed to 1 mg/mL.

#### 3.5.2. Determination of Minimum Inhibitory Concentration (MIC) by the Microdilution Method

The three bacterial strains used to analyze the zone of inhibition were also used to determine the minimum inhibitory concentration of guava leaf extracts against the growth of selected bacteria by broth dilution method [59] with the concentrations, 950 to 0.928 µg/mL in two-fold serial dilution. Amoxicillin/ciprofloxacin were used as the standard antibiotic drugs.

### 3.6. Statistical Analysis

All the assays of biological activities were performed in triplicate and their average was calculated. The data were expressed as the mean ± standard deviation using MS Office Excel (Ver. 16). The enzyme inhibitory and free radical scavenging effects were represented as IC_50_ as well as a percentage of inhibition. The graphical illustrations of the data were executed with the software GraphPad Prism (Ver. 9.3.1). Correlation and Principal component analysis (PCA) analysis between the nutritional components and reported biological activities were evaluated by R (Ver. 4.3.0) programming software.

## 4. Conclusions

In a rapidly developing world, the majority of people show the least concern towards food hygiene and the emergence of diseases is also high, causing health issues. In this regard, scientific communities need to pay more attention to developing drugs from natural sources which may have scanty side effects. *Psidium guajava* is considered to be an inevitable source of various phytocompounds that have high potential with countless biological activities. This present study scrutinized the nutritional profile and biological potentialities of the leaf extract from different cultivars of *P. guajava*. Elements like calcium, magnesium, and nitrogen recorded higher ratios. The methanolic extract of the cultivars showed notable quantities of phenol and flavonoids and also recorded proficient results over biological assays. Further studies of the chemical profiling of the dominant extract and cultivars can be performed in order to identify the biologically active compound that is accountable for various health benefits and their usage can be encouraged as an additive for food supplements.

## Figures and Tables

**Table 1:** Elemental analysis of four *Psidium guajava* cultivars’ leaf extract.

Parameters	Allahabad Safeda	Surka Chitti	Karela	Lucknow-49
Total nitrogen (mg/100 g)	1730 ± 1.09	2800 ± 1.27	1450 ± 1.41	2350 ± 1.43
Total phosphorus (mg/100 g)	170 ± 0.54	160 ± 1.08	210 ± 1.09	320 ± 1.12
Total potassium (mg/100 g)	480 ± 0.67	1140 ± 1.16	700 ± 1.26	1070 ± 1.28
Total sodium (mg/100 g)	20 ± 0.4	14 ± 0.17	24 ± 0.04	16 ± 0.14
Total calcium (mg/100 g)	2560 ± 1.12	2720 ± 1.43	2720 ± 1.37	1440 ± 1.39
Total magnesium (mg/100 g)	1340 ± 1.38	860 ± 0.49	1440 ± 1.24	1150 ± 1.42
Total iron (mg/100 g)	51.6 ± 0.76	15.9 ± 0.81	22.5 ± 0.50	44.8 ± 0.19
Total zinc (mg/100 g)	0.46 ± 0.32	0.32 ± 0.11	0.48 ± 0.07	0.32 ± 0.07
Total copper (mg/100 g)	0.51 ± 0.09	0.368 ± 0.29	0.52 ± 0.81	0.667 ± 0.26
Total boron (mg/100 g)	4 ± 0.10	1 ± 0.03	3 ± 0.02	3 ± 0.04

**Table 2:** Minimum inhibitory concentration of the leaf extracts of *P. guajava* cultivars.

Cultivars	Extracts	Minimum Inhibitory Concentration (µg/mL)		
		Gram-Positive Bacteria	Gram-Negative Bacteria	
		*B. subtilis*	*P. vulgaris*	*P. aeruginosa*
Surka chitti	Ethyl acetate	118.75	950	59.38
	Methanol	237.5	950	118.75
	Aqueous	237.5	950	118.75
	Standard	15	0.23	0.05859
Allahabad safeda	Ethyl acetate	237.5	475	14.84
	Methanol	118.75	118.75	29.69
	Aqueous	475	118.75	0
	Standard	0.9375	>60	0.11719
Karela	Ethyl acetate	59.38	118.75	59.38
	Methanol	118.75	118.75	59.38
	Aqueous	950	950	950
	Standard	0.9375	>60	0.11719
Lucknow-49	Ethyl acetate	237.5	475	950
	Methanol	3.71	475	59.38
	Aqueous	>950	237.5	950
	Standard	15	0.23	0.05859

## Data Availability

Data is contained within the article.
